# Conduit design with expanding diameter for enhanced flow

**DOI:** 10.1038/s41598-023-36165-6

**Published:** 2023-06-23

**Authors:** Javad Hashemi, Hunter Peeples, Riley Kuykendall, Seshadri Raju, Ghassan S. Kassab

**Affiliations:** 1 California Medical Innovation Institute, San Diego, CA USA; 2 The RANE Center, 971 Lakeland Drive, Suite 401, Jackson, MS 39216 USA

**Keywords:** Computational biology and bioinformatics, Engineering

## Abstract

Conduits are commonly used for treating lesions in arteries and veins. The conventional stents are cylindrical in shape, which increases flow resistance with length. This study presents a design of stents and conduits where the conduit caliber expands gradually to reduce resistance while avoiding flow separation. Inflow was provided from a header tank at two different pressures (i.e., 10 and 25 mm Hg pressure) into a cylindrical or expanding conduit. The initial conduit calibers were 2-, 3-, 4-, and 5-mm and 160-, 310-, and 620-mm lengths in each case. The flow rates of expanding caliber conduits (at a rate of r^4–6^/cm where r is the initial conduit radius) were compared to traditional cylindrical conduits of constant radius. The expanded caliber yields a significantly increased flow of 16–55% for R^4^/L expansion, 9–44% for R^5^/L expansion, and 1–28% for R^6^/L expansion. Simulated flow models using computational fluid dynamics (CFD) were used to validate and expand the experimental findings. Flow separation was detected for certain simulations by flow pathlines and wall shear stress (WSS) calculations. The results showed that a caliber expansion rate of r^6^/cm is the optimal rate of expansion for most potential applications with minimum flow separation, lower resistance, and increased flow.

## Introduction

A conduit with expanding diameter is called a diffuser, which serves to increase the pressure of a compressible fluid (i.e., gas) along the length of the diffuser. A diffuser is used for condensing gas in jet engines, turbo engines and air pumps that have turbulent flow in such applications as nozzles of jet engines^1^. In the human body, the veins have an expanding caliber similar to a diffuser that conducts blood (incompressible fluid) for a different function, i.e., to minimize resistance to flow. This is critical as the veins represent the tail end of the circulation where most of the motive energy generated by the heart has been expended. The remaining residual energy (< 5%) should be preserved to complete the flow circuit back to the heart^2,3^. A diffuser type of expanding conduit design may be superior to the traditional cylindrical design in functionality with better flow and a lower expenditure of energy in Poiseuille flows.

Attempts at improving basic conduit design to augment flow have been surprisingly sparse, considering that the traditional cylindrical design was used for several millennia. Currently, available methods to augment flow include booster pumps, storage tanks (additional energy), and devices such as valves and surge tanks to optimize flow and suppress transients. Strategies such as siphons and bypasses have also been used to minimize energy waste from flow transformation (pressure head to velocity and vice versa). Conduits with polished surfaces minimize frictional losses; a smooth profile without bends, local expansions, and constrictions may reduce minor losses^4,5^.

Here, a new design with a gradually expanding caliber is proposed, showing a substantial reduction in flow resistance over the cylindrical design. The flow improvement is made possible by modulating caliber with a length such that overall flow resistance is reduced. The challenge is that expanding caliber may create flow separation. Flow separation occurs in different fluidic systems, which impede flow and degrade device performance.

Recent advances in experimental and numerical fluid mechanics have led to greater elucidation of pathological events at the inner wall of vessel (i.e., endothelium). At the core of it, a low WSS, caused mainly by flow separation, is a common denominator^6^. These studies were performed by three-dimensional reconstruction of artery segments with subsequent numerical flow simulation studies. Computational fluid dynamics (CFD) methods combined with three-dimensional vessel reconstructions based on medical imaging allow the calculation of desired hemodynamic parameters under realistic boundary conditions with high resolution^6–10^. Numerical flow simulations were used because accurate in vivo measurements of velocity profiles in the arteries are not feasible.

In the present study, a new conduit design is proposed with expanding diameter that improves flow with minimum flow separation. In the process, we use both bench test and CFD for characterizing conduit flow and optimize the device based on WSS and pressure drop. A comparison of the flow patterns obtained with the new design and the traditional cylindrical conduit is reported in a bench model and CFD simulation. The CFD predicted possible areas of flow separation in some dimensions of the gradually expanding profile, which can be avoided with proper design. Beneficial profiles with improved flows compared to the traditional cylindrical design can be identified.

This novel design may find use in medicine where prosthetic conduits are used to transmit low volume/velocity flows occurring due to specific anatomic location or disease. There are numerous potential applications for the design in engineering products where conduits are used to carry various fluids such as fuel, lubricants, coolants etc. Drains using the new design may be more efficient with less clogging.

## Methods

### Theoretical basis of expanding conduit profile

The Poiseuille equation expresses laminar flow:

$$\mathrm{F}=\mathrm{\Delta P}\times \frac{\uppi }{8}\times \frac{{\mathrm{r}}^{4}}{\mathrm{l}}\times 1/\upeta .$$

Flow for a given pressure gradient is governed by three coefficients—known respectively as the numeric, geometric, and viscosity factors. The numeric factor is constant, and viscosity is also a constant for a Newtonian fluid (saline used in the experiments). The geometric factor is the variable (resistance) regulating flow in laminar flow. The geometric factor is notable because it appears in the fourth power as the numerator. The length of the conduit is less consequential in the first power in the denominator. The flow in traditional cylindrical conduits gradually declines with length due to a linear additive increase in flow resistance. We hypothesized that the linear increment in resistance could be offset by a relatively smaller increase in radius due to its higher power effect. The ideal offset is to increase the radius per each successive 1 cm length so that r^4^/l remains constant and equal to the first cm of the conduit. An expanding tube has a gradually expanding profile over its length. An expansion rate of r^4^/cm will double its radius at 16 cm in length. A more practical formulation is to keep r^5^/l or even r^6^/l constant over the length of the conduit yielding a more gradual conduit expansion than keeping r^4^/l constant. This will yield longer tube lengths before the radius doubles (see Table 1). The flow conductive performance will be less than the constant r^4^/l formulation but still better than a uniform cylinder, and flow separation may also be less pronounced. The longer length conduits may be needed for certain applications. Note that the rate of conduit expansion at R^5^ and R^6^ counterintuitively yields a more gradual expansion rate than R^4^ (Table 2). This is because the incremental conduit length (L) is distributed over larger numbers associated with R^5^ and R^6^ compared to R^4^. The present CFD simulations are limited to steady-state straight-line non-pulsatile flow. The formulation of the field equations for the CFD simulations are outlined in the “Appendix”.

**Table 1:** Initial and end diameter of uniform cylindrical and expanding test conduits.

Geometric factor	Initial diameter (mm)	Ending diameter at L = 160 mm (mm)	Ending diameter at L = 310 mm (mm)	Ending diameter at L = 620 mm (mm)
Cylinder: $\mathrm{r}$	4	4.00	4.00	4.00
	6	6.00	6.00	6.00
	8	8.00	8.00	8.00
	10	10.00	10.00	10.00
$\frac{{\mathrm{r}}^{4}}{\mathrm{L}}$	4	8.00	9.44	11.22
	6	12.00	14.16	16.84
	8	16.00	18.88	22.45
	10	20.00	23.60	28.10
$\frac{{\mathrm{r}}^{5}}{\mathrm{L}}$	4	6.96	7.95	9.13
	6	10.45	11.92	13.70
	8	13.93	15.90	18.26
	10	17.41	19.87	22.83
$\frac{{\mathrm{r}}^{6}}{\mathrm{L}}$	4	6.35	7.09	7.96
	6	9.52	10.63	11.94
	8	12.70	14.18	15.92
	10	15.87	17.72	19.89

**Table 2:** Expanding stent caliber configuration for Iliac vein stent (initial diameter = 14 mm).

Length (cm) L	R^4^/L radius (mm)	R^5^/L radius (mm)	R^6^/L radius (mm)
0	14.00	14.00	14.00
1	14.00	14.00	14.00
2	16.65	16.08	15.71
3	18.43	17.44	16.81
4	19.80	18.47	17.64
5	20.93	19.32	18.31
6	21.91	20.03	18.87
7	22.77	20.66	19.36
8	23.55	21.22	19.80
9	24.25	21.73	20.19
10	24.90	22.19	20.55
11	25.50	22.62	20.88
12	26.06	23.01	21.18
13	26.58	23.38	21.47
14	27.08	23.73	21.73
15	27.55	24.06	21.99

Expansion factors of R^5^ and R^6^ yield a smaller rate of conduit expansion compared to R^4^.

### Fabrication of experimental conduits

The conduit designs were designed using engineering software (Autodesk, Inc.; San Rafael, CA) and fabricated in a commercial 3D printer (Stratasys; Eden Prairie, MN).

#### Flow test bed

**Figure 1:**
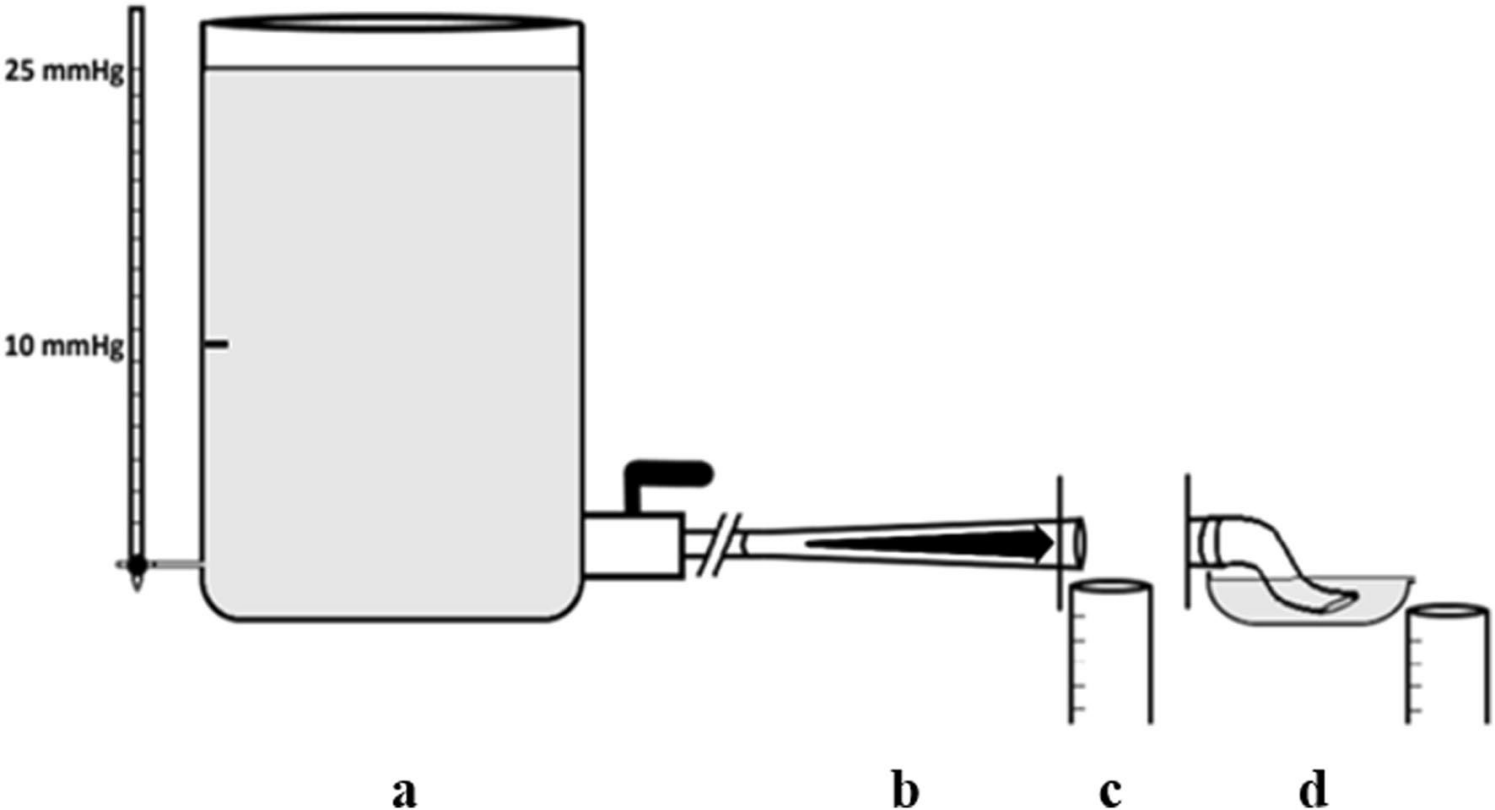
Basic conduit flow model. (**a**) Upstream pressures of 10- and 25-mm Hg were generated using the header tank. (**b**) The various conduits were inserted into the basic conduit flow model. (**c**) Volume released into the discharge tank in 1 min was used to calculate the flow rate of each conduit. (**d**) In some experiments, a partially closed system of drainage or Penrose “air trap” was used.

The basic flow model consisted of a header tank with outflow controlled by a calibrated ball valve (Fig. 1). The ball valve was kept open at the same setting for all flows. The various conduits were connected to the ball valve. Conduit outflow was open to the atmosphere (open system) and was allowed to drain into a graduated cylinder for timed measurement (cc/min). In some experiments, a partially closed drainage system was used: the conduits were connected to a short Penrose drain (Diameter = 3.5 cm; Length = 13 cm) discharging under the fluid level in a shallow pan before emptying into the output cylinder (Fig. 1, option D). The system prevented air from entering the conduit, possibly inhibiting flow separation. The system was filled with a 2:3 mixture of glycerol and water with a viscosity of 0.004 kg/m/s. Each flow measurement is an average of 5 ‘runs’.

#### CFD modeling

**Figure 2:**
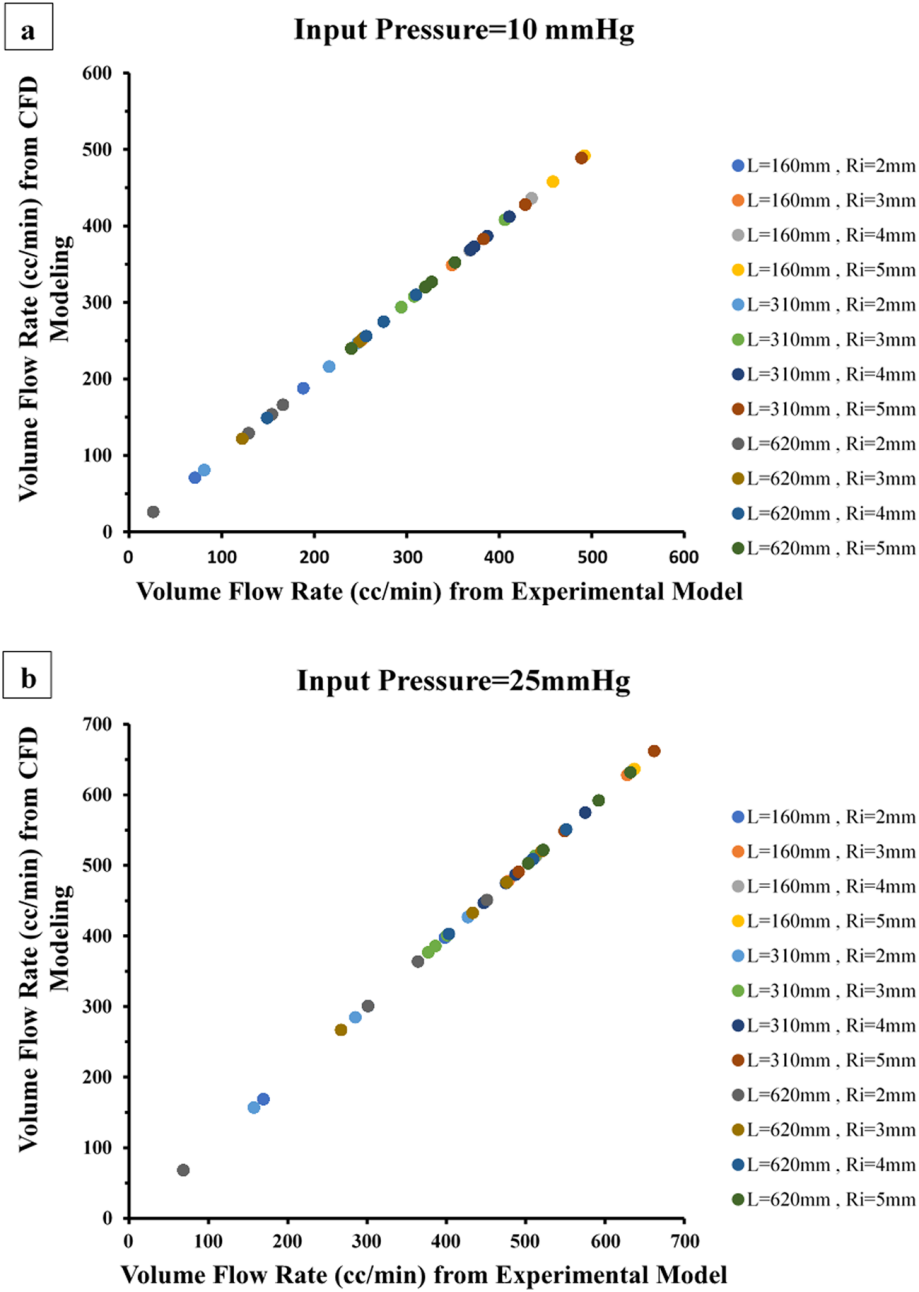
Flow rate comparison between experiments and simulations for model validation with input pressures (**a**) 10 mmHg and (**b**) 25 mmHg. CFD flows are nearly identical to the mean flows obtained in the mechanical bench model.

Flow conduit was simulated based on CFD using a Newtonian Single-phase 3D model (see “Appendix”). The single-phase 3-D Eulerian equation using a laminar viscous model was solved with ANSYS Fluent 21.0 (Ansys Inc; Canonsburg, PA). The blood viscosity (0.004 kg/m/s) defines as Newtonian model^11^. A mixture density for blood of 1040 kg/m^3^ was used. Geometries for each CFD run to simulate conduits in the experimental model were derived from Design Modeler 21.0. Mesh ANSYS 21.0 was used to generate unstructured cell mesh for the geometries, and mesh sensitivity analysis determined an optimal node count for volume flow rate. Flow separation analysis was based on model-derived pathlines and calculated thresholds using WSS and pressure. The inlet boundary condition was a constant pressure as represented in Table 3. The outlet boundary condition was an outlet pressure. The model was validated by comparing computed and experimental volume flow rate (Fig. 2).

**Table 3:** Mean conduit flow rate at Input Pressure of 10 mmHg and 25 mmHg when R, R^4^/L, R^5^/L, and R^6^/L are held constant (no air-trap).

Conduit length (mm)	Initial radius (mm)	Constant radius flow (cc/min)	Constant R^4^/L flow (cc/min) (% improvement)	Constant R^5^/L flow (cc/min) (% improvement)	Constant R^6^/L flow (cc/min) (% improvement)
Input pressure = 10 mmHg					
160	2	71	188 (+ 165%)****	–	–
	3	251	349 (+ 39%)**	–	–
	4	368	435 (+ 18%)**	–	–
	5	458	492 (+ 7%)	–	–
310	2	81	294 (+ 263%)****	216 (+ 167%)****	247 (+ 205%)****
	3	294	406 (+ 38%)***	321 (+ 9%)	308 (+ 5%)
	4	373	411 (+ 10%)	387 (+ 4%)	369 (− 1%)
	5	428	489 (+ 14%)***	428 (0%)	383 (− 11%)
620	2	26	166 (+ 538%)****	154 (+ 492%)***	129 (+ 396%)***
	3	122	253 (+ 107%)***	249 (+ 104%)***	240 (+ 97%)**
	4	149	310 (+ 108%)***	275 (+ 85%)***	256 (+ 72%)****
	5	240	352 (+ 47%)**	327 (+ 36%) **	320 (+ 33%)*
Input pressure = 25 mmHg					
160	2	169	398 (+ 136%)****	–	–
	3	478	628 (+ 31%)***	–	–
	4	513	636 (+ 24%)**	–	–
	5	550	637 (+ 16%)**	–	–
310	2	157	427 (+ 172%)****	301 (+ 92%)**	285 (+ 82%)*
	3	401	512 (+ 28%)**	377 (− 6%)	386 (− 4%)
	4	475	575 (+ 21%)*	447 (− 6%)	487 (+ 3%)*
	5	549	662 (+ 21%)***	491 (− 11%)*	520 (− 5%)****
620	2	68	451 (+ 563%)****	364 (+ 435%)****	301 (+ 343%)
	3	267	520 (+ 95%)****	476 (+ 78%)****	433 (+ 62%)**
	4	403	551 (+ 37%)****	504 (+ 25%)**	509 (+ 26%)****
	5	503	632 (+ 26%)**	592 (+ 18%)***	522 (+ 4%)

*P < 0.05 vs. constant radius flow.

**P < 0.01 vs. constant radius flow.

***P < 0.001 vs constant radius flow.

****P < 0.0001 vs. constant radius flow.

### Statistical analysis

A commercial software package was used (GraphPad Prism, San Diego; CA.). Chi-square, paired, and unpaired two-tailed t-tests were used as appropriate to compare flows. A p-value < 0.05 was considered significant.

## Results

### Test bed

The flow rates in the test bed of expanding caliber conduits (r^4 to 6^) compared to traditional cylindrical conduits are shown in Table 3. In some runs, the outflow stream was observed to be separated from part of the tube outlet circumference, suggesting flow separation from the wall. This problem was reduced when the Penrose air trap was used (Table 4). Overall, the expanded caliber yields a significantly higher flow ranging from 16 to 55% for R^4^/L expansion, 9–44% for R^5^/L expansion, and 1–28% for R^6^/L expansion.

**Table 4:** Mean conduit flow rate with and without Penrose air-trap (conduit length = 310 mm).

Initial radius (mm)	Cylinder: R flow^a^ (cc/min)	R^4^/L flow (cc/min, %)	R^4^/L + air-trap flow (cc/min, %)	R^5^/L flow (cc/min, %)	R^5^/L + air-trap flow (cc/min, %)	R^6^/L flow (cc/min, %)	R^6^/L + air-trap flow (cc/min, %)
Input pressure = 10 mmHg							
3	294	406 (+ 38%)	456 (+ 55%)***	321 (+ 9%)	424 (+ 44%)***	308 (+ 5%)	376 (+ 28%)**
4	373	411 (+ 10%)	450 (+ 21%)***	387 (+ 4%)	444 (+ 19%)***	369 (− 1%)	389 (+ 4%)
5	428	489 (+ 14%*)	510 (+ 17%)**	428 (0%)	467 (+ 9%)*	383 (− 11%)	443 (+ 4%)
Input pressure = 25 mmHg							
3	401	512 (+ 28%)	570 (+ 42%)****	377 (− 6%)	553 (+ 38%)****	386 (− 4%)	449 (+ 12%)*
4	475	575 (+ 21%)	602 (+ 27%)**	447 (− 6%)	566 (+ 19%)**	487 (+ 3%)	505 (+ 6%)*
5	549	662 (+ 21%)	638 (+ 16%)**	491 (− 11%)	602 (+ 10%)**	520 (− 5%)	553 (+ 1%)

*P < 0.05 vs. constant radius flow.

**P < 0.01 vs. constant radius flow.

***P < 0.001 vs constant radius flow.

****P < 0.0001 vs. constant radius flow.

^a^Flow separation did not occur in the constant radius conduits; Penrose air-traps did not affect these flows.

### CFD simulation

**Figure 3:**
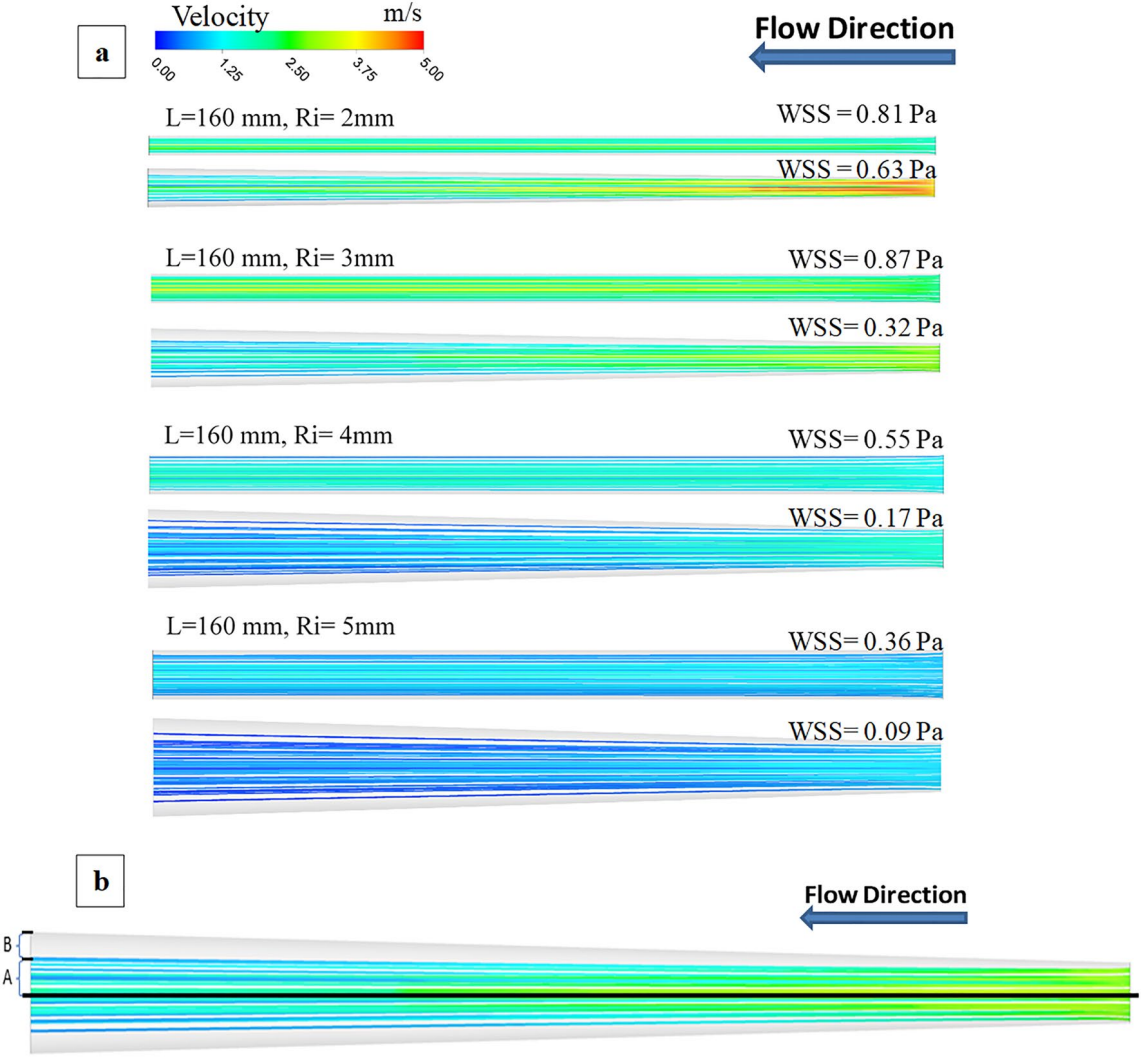
(**a**) Velocity pathlines (m/s) for R^4^ expanding conduit as compared to cylinder of given radius (Ri); and same inlet pressure of 25 mmHg. The area average wall shear stress (WSS) is listed for each of the simulations for comparison. (**b**) Sample flow pathlines to visualize secondary flow (B) for detection of flow separation. When the secondary flow is higher or equal to 33% of the outlet radius and where area averaged WSS reduction (A) is about 70%, the flow is fully separated.

Since wall flow separation may be difficult to visualize in the experimental setup, we used CFD to simulate all the cases performed in the bench model (Fig. 3a,b). CFD simulation was validated by comparing mean conduit flow measured experimentally with CFD simulation runs (Fig. 2a,b); experimental and simulation flows were nearly identical. The CFD distributions of the WSS for the simulations are shown in Table 5, which displays the fully separated flow cases with red color and non-significant flow separation cases with blue. For most diameter/length combinations, an expansile rate of r^6^/cm appears to yield the most coherent flow. A greater expansile rate of r^4^/cm may be possible for small diameter conduits. Figure 3 shows areas with very low WSS, marked in blue, corresponding to the flow separation regions. The color scale between 0 and 5 Pa was selected to underline the regions with very low WSS.

**Table 5:**
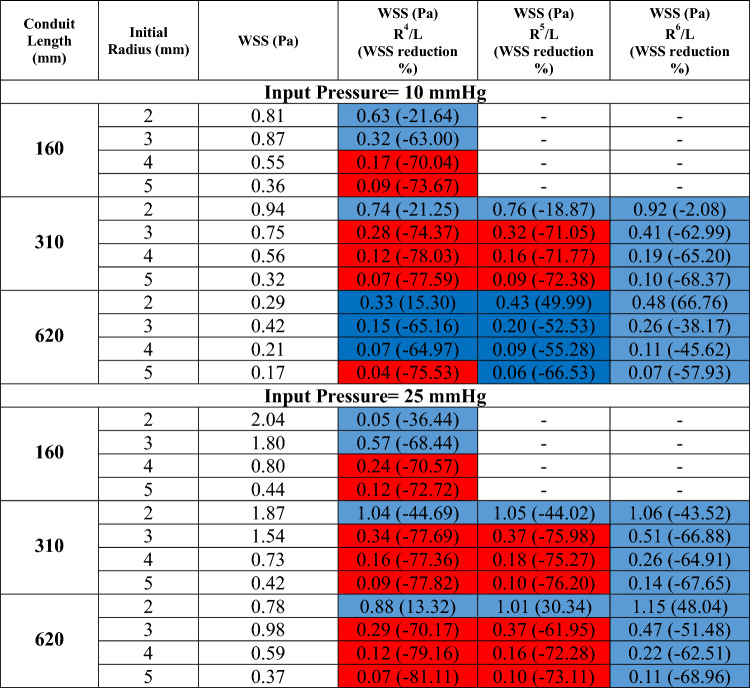
WSS when R, R4/L, R5/L, and R6/L are held constant, and the fully separated flow highlighted with red color and non-significant flow separation highlighted with blue color.

The results show that flow detachment occurs in low WSS, and high-pressure drop regions. Figure 3a shows the velocity pathline zones with normal flow and secondary flow seen in Fig. 3b. Based on the analysis if the secondary flow (B) is higher or equal to 33% of the outlet radius and where WSS reduction is about 70%, then flow is fully separated. The results showed that the expansion rate with R^4^/cm increased flow detachment for conduits with 4 and 5 mm as initial radius. R^6^/cm is a better expansion rate for small conduits with an initial 2- and 3-mm radius. In addition, increasing the pressure boundary condition increased possible flow detachment. The results also showed that pressure drop and WSS have an inverse relationship. The cases with the fully separated flow have a high-pressure drop, while WSS was significantly reduced.

## Discussion

The traditional design of conduits used in medicine and engineering are cylinders of constant caliber. A fundamental design change where the caliber expands gradually with length is proposed. Such a profile exhibits greater flow with lesser resistance in a mechanical bench model and is validated by CFD. A conduit expansion rate where r^4^/cm remains constant is the theoretical optimum, but the caliber will double by 16 cm conduit length. This may be practical for certain medical applications, such as short prosthetic grafts. A more gradual expansile rate of r^5^/cm or even r^6^/cm will yield more useful longer-length conduits before the diameter doubles. While somewhat less efficient than r^4^/cm expansion, they are still superior to traditional cylindrical conduits. These may be more appropriate for engineering applications.

The slope of the expansion dictates the propensity of flow detachment which is counter-productive in terms of flow optimization (i.e., increases dissipation and flow resistance); i.e., too abrupt of an expansion will cause flow detachment and increased resistance to flow despite the increased in diameter. Hence, there is a compromise between the rate of conduit diameter expansion and the prevention of flow detachment, as demonstrated in this study. More complex conduit designs may involve expansive length elements followed by cylindrical or converging elements to re-attach the flow. An entrance length (i.e., the distance through which the velocity will be redistributed from blunt or uniform to parabolic laminar profile) is known to be proportional to Reynold’s number. Based on these design principles, various conduits can be designed based on dimensional specification and tested on bench and CFD simulations to yield the desired high-flow conduits.

Accretive manufacturing (3-D) makes it much easier to fabricate expanding caliber grafts and conduits for biological or mechanical use. Current findings suggest that up to ≈16 cm is possible for conduit designs keeping r^4^/L constant. Up to 64 cm in length appears possible for conduits keeping r^5^/L constant. Some potential applications for the new design with expanding diameter in Medicine and Engineering are briefly discussed below.

### Medical applications

#### Drains and shunts

For centuries, prosthetic drains have been used to drain blood or serum collections after surgery. Early models were made of rubber, while more recently, prosthetic conduits made of silicone or polymer have been used to drain various body fluids. The drains are used for cerebrospinal fluid, ascites, or pleural effusions into adjacent body cavities or the atrium for absorption (e.g., Denver shunt, LeVeen shunt). These are low-pressure systems (≤ 10 mm Hg) with low flow velocity. It is a struggle to keep the drain conduits patent and functional. Vacuum assistance at the downstream end of the conduit (Graduated ‘underwater’ suction or compressed bellows drainage chamber) is often used when the drainage is to the outside. Such vacuum assistance may be counterproductive, sucking tissue or coagulated debris plugging the drain. A manually compressible inline pump is sometimes provided (e.g., Denver shunt) for internal drainage. These systems may function better if the conduit resistance can be reduced.

#### Conduits and stents

Prosthetic conduits and stents made of silicone or polymer are commonly used to treat strictures in the gastrointestinal system (e.g., esophagus, biliary tract, etc.) and the urinary tract. The biliary and ureteral stents are typically small caliber conduits prone to blockage and resulting infection. Better functionality may be possible with the expanding diameter design. Metal stents are used in a variety of locations. Attempts have been made to improve the hemodynamic performance of vascular stents by focused stent strut design^12^. The expanding diameter design may not be appropriate in locations where the expanded end may result in a size mismatch to the native vessel. This size mismatch is less of a problem in veins where the natural scaling is an increase in size along the flow direction. Also, venous stents get rapidly covered by fibrous tissue when strut design is no longer a flow influencer^13,14^. The covered stent becomes a scaffold for remodeling the stented vein. Good apposition of the stent to the stented structure/vessel can be achieved by a variety of techniques such as balloon dilatation before and after stent deployment particularly if the native structure/vessel is adequately compliant to accommodate the self-expanding stent design.

#### Venous grafts

Synthetic grafts function well in the arterial system due to the high input pressure (≈ 100 mm Hg). Grafts in the venous system are prone to thrombosis because the input pressure is much lower (≈ 25 mm Hg)^15^. An adjunctive arterio-venous fistula is often created to provide large volume flow in an effort to maintain patency. A synthetic graft with an expanding profile may improve patency.

### Dialysis grafts

Inadequate flow for adequate dialysis is a common problem with legacy dialysis grafts. Low flow prosthetic grafts provide inefficient dialysis and are at risk of thrombosis^16^. A 6 mm diameter 25 cm length dialysis graft is commonly used in clinical practice. A 6 mm diameter 31 cm length conduit yields 294 and 401 cc/min (10- and 25-mm Hg input pressure) in the test bed. In comparison, the expanding configurations yield increases in flow up to 55% and 42% for 10 and 25 mmHg input pressure, respectively. Quantitative flows in patients may be different because of higher input pressures. While quantitative duplication of clinical flows in the test bed is not offered, the relative flow advantage in the tested range is a useful indicator.

### Mechanical applications

Conduits are the circulating system of modern industry that includes engines, machinery, electronics, and evolving ‘green’ systems based on batteries. These conduits carry fluid of all kinds: Fuel, coolants, lubricants, hydraulic fluids, etc. The expanded configuration conduit may be more efficient than the traditional cylindrical design in these applications where the fluid/liquid behaves subject to Poiseuille Flow Equation. These industrial applications have an advantage over human biologic conduits. There is greater flexibility in designing adequate power input to maintain flow; the base caliber/length of the conduit can be designed for optimal performance without turbulence. A partially closed flow system like the one used in the flow model may be required to prevent flow separation in the conduit. In mechanical systems, the ability to adjust input pressure, base caliber, and length to suit the flow demand is an advantage over biologic applications. The CFD method is a powerful tool to design application specific conduits to provide improved flow over the traditional cylindrical design.

## Conclusion

A novel expanded profile conduit is described that has a slowly expanding diameter to offset flow resistance that increases linearly with the length of the conduit. This is based on Poiseuille law, where the radius enters the flow equation as the numerator in the fourth power while length is on the denominator side in the first power. Hence, the increase in diameter to offset increasing flow resistance with length is smaller by order of magnitude. The expanding diameter design exhibits superior flow to the traditional cylindrical design by reducing overall flow resistance. This can be demonstrated in mechanical flow systems and CFD. The new design conserves energy and has a wide field of applications in medicine and engineering where non-turbulent conduit flow occurs. CFD is a powerful tool for designing conduits to suit individualized specifications.

## Data Availability

The datasets generated during and/or analyzed during the current study are available from the corresponding author on reasonable request.

## Appendix

Computational fluid dynamics (CFD) solves governing conservation equations that depend on the individual system. The equation for energy conservation can be included in addition to the conservation equations of mass and momentum. Continuity is described for the conservation equation of mass by:

1 $$\frac{\partial \rho }{\partial t}+\left(\nabla \cdot \rho v\right)={S}_{m},$$

where ρ is density, v is velocity, and Sm is a source term. The momentum conservation equation is given by:

2 $$\frac{\partial }{\partial t}\left(\rho v\right)+\nabla \cdot \rho vv=-\nabla p-\left[\nabla \cdot \tau \right]+\rho g.$$

The viscous momentum flux tensor τ represents shear stress and is given by:

3 $$\tau =-\mu \left[\nabla v+{\left(\nabla v\right)}^{T}\right]+\frac{2}{3}\mu \left(\nabla \cdot V\right)\delta .$$

Assuming constant viscosity (for saline used in the experiments) and fluid density, substitution of τ in the conservation equation of momentum and adjustment gives the Navier–Stokes equation:

4 $$\rho \left(\frac{\partial V}{\partial t}+v\cdot \nabla v\right)=-\nabla p+\mu \nabla 2v+\rho g.$$

CFD provides a solution to the mass conservation and Navier–Stokes equations by approximating the continuous variables in the partial differentials using discrete analogs and utilizing a geometric mesh or grid discretized domain. For CFD, flow variables are defined at every point on the domain for continuous domains. An accurate solution may be obtained when each node interacts with adjacent nodes predictably. Discretized results approach analytical results when the grid resolution (i.e., mesh count) is sufficient.
